# A systematic review on the health effects of fermented wheat germ extract with emphasis on cancer

**DOI:** 10.3389/fnut.2025.1677464

**Published:** 2025-11-12

**Authors:** Muzeyyen Berkel Kasikci, Aline Issa, Nurcan Baglam, Emine Dincer, Amaury Gérard, Sumeyye Kabakci, Vasfiye Hazal Özyurt, Zsolt Zalán, Taner Şar, Burcu Gündüz Ergün, Christophe Chassard, Smilja Praćer, Guy Vergères, Mustafa Guzel

**Affiliations:** 1 Department of Food Engineering, Manisa Celal Bayar University, Manisa, Türkiye; 2 Department of Nursing and Health Sciences, Notre Dame University– Louaize, Zouk Mikael, Lebanon; 3 Department of Nutrition and Dietetics, Sivas Cumhuriyet University, Sivas, Türkiye; 4 Brewing and Food Industry (BrAlim) Research Unit, LABIRIS, Brussels, Belgium; 5 National Food Reference Laboratory, Ministry of Agriculture and Forestry, Ankara, Türkiye; 6 Department of Gastronomy and Culinary Arts, Mugla Sitki Kocman University, Mugla, Türkiye; 7 Institute of Food Science and Technology, Hungarian University of Agriculture and Life Sciences Research, Budapest, Hungary; 8 Swedish Centre for Resource Recovery, University of Borås, Borås, Sweden; 9 Health Biotechnology Centre of Excellence for Joint Research and Application (SABIOTEK), Yildiz Technical University, Istanbul, Türkiye; 10 Department of Molecular Biology and Genetics, Faculty of Arts and Sciences, Yildiz Technical University, Istanbul, Türkiye; 11 UCA, INRAE, VetAgro Sup, UMRF 0545, Aurillac, France; 12 Institute for Biological Research Sinisa Stankovic, National Institute of Republic of Serbia, University of Belgrade, Belgrade, Serbia; 13 Research Division Microbial Food Systems, Agroscope, Berne, Switzerland; 14 Department of Food Engineering, Hitit University, Corum, Türkiye

**Keywords:** fermented wheat germ extract, fermentation, novel foods, benzoquinones, cancer

## Abstract

Fermented wheat germ extract (FWGE) is one of the few fermented food products listed in the EFSA novel food Catalogue. It is derived from wheat germ, a by-product of wheat processing, through fermentation with *Sacchoromyces cerevisiae*. The most widely studied and patented form of FWGE is marketed as Avemar (also referred to as MSC), standardized to contain methoxy-substituted benzoquinones. Given its predominant use in research, this systematic narrative review focused primarily on FWGE use within the medical application. The objective of this review was to systematically evaluate the functionality and potential health benefits of FWGE, following the Study Protocol-S8 developed under COST Action CA20128 – PIMENTO and registered on the Open Science Framework (https://osf.io/fq53j/). A systematic literature search of human studies was conducted through PubMed, Scopus, and Cochrane Library. Studies involving adult participants who received FWGE interventions were included, with primary clinical endpoints selected according to the main indications for FWGE. Additional outcomes were reported when available. Out of the 51 records identified by the literature search, six studies met the inclusion criteria. Data from these studies were extracted and synthesized in summary tables. Supplementary information on the functionality of FWGE was retrieved through a non-systematic search of animal and *in vitro* studies. Furthermore, this review highlights the potential bioactive constituents of FWGE, particularly benzoquinones, peptides, and phenolic compounds, as mediators of its anticancer and anti-inflammatory properties. Among its proposed mechanisms, FWGE may suppress cancer cell proliferation by disrupting the glucose-related metabolic pathways. While the findings suggest that FWGE may possess therapeutic potential, especially in oncology, the strength of evidence remains limited. Of the six included human studies, only four employed proper control groups and only two demonstrated high methodological quality. As such, the current evidence base is insufficient to draw definitive conclusions, and well-designed clinical trials are needed to strengthen this evidence. Moreover, future research should also explore the development of novel FWGE formulations with enhanced bioactive profiles, optimized by modulating fermentation conditions, including such as microbial strain, pH, temperature, and duration.

## Introduction

From yogurt in the Middle East to Korean kimchi, Fermented Foods (FFs) not only reflect culinary variations and an appealing taste but also embody the cultural heritage of a region and represent the practices performed by the community during preparation and consumption (1). The nutritional benefits of FFs frequently surpass those of nonfermented foods. This can be attributed to the fermentation process (2) Furthermore, with the global population on the rise and increasing concerns about food security, FFs offer sustainable alternatives to conventional food production practices (3).

Historically, FFs have been an essential component of human nutrition. Initially consumed for their extended shelf life, ease of storage, and low-cost production, many of these foods are now valued primarily for their health benefits (4). This is primarily due to high nutritional value and bioactive compounds such as vitamins, peptides, and organic acids. Various FFs offer varying health benefits (4, 5). These benefits vary depending on the composition of the final product that were formed during fermentation. Moreover, the probiotic microorganisms that might be present in some FFs help maintain a healthy gut microbiota, therefore supporting digestive functions and improving immune responses (6–8). In addition to the previously mentioned benefits, studies have shown that some FFs may lower cholesterol levels, protect against pathogens, and reduce the risk of diseases such as cancer, osteoporosis, diabetes, obesity, allergies (5, 9, 10), and gastrointestinal, chronic, and cardiovascular diseases (1, 6–9, 11), in addition to enhanced immune responses (12–14).

Fermented Wheat Germ Extract (FWGE) stands out as a compelling showcase for novel FFs, representing a unique intersection of traditional fermentation techniques and modern scientific understanding. FWGE is produced through the fermentation of wheat germ, the nutrient-rich embryo of the wheat kernel, using *Saccharomyces cerevisiae*, also known as baker’s yeast (15, 16). This process enriches the end-product with biologically active compounds, particularly benzoquinones, which are believed to contribute to its potential health benefits (16) Benzoquinones are naturally occurring quinones present in plants, fungi, bacteria, and animals, where they play key roles in electron transport, oxidative phosphorylation, and other bioenergetic processes. Increasing evidence indicates that several benzoquinones exhibit strong antioxidant, anti-inflammatory, and anticancer properties, with many studies investigating the mechanisms underlying these effects. While their antioxidant capacity forms the basis of much of their biological activity, the specific outcomes are influenced by the surrounding biological microenvironment. A major advantage of benzoquinones is their relative ease of synthesis and chemical modification, which enhances their potential as scaffolds for developing novel therapeutic agents (17, 18). Its capacity to undergo redox cycling in the presence of ascorbate further strengthens its anticancer activity. Importantly, fermentation is essential for generating these active compounds, as microbial *β*-glucosidase converts glycosylated precursors in wheat germ into free benzoquinones with demonstrated antimicrobial and immunomodulatory properties (17, 18).

The development of FWGE has its roots in the 1990s, when Hungarian chemist Máté Hidvégi pioneered its creation (19). This historical context underscores the relatively recent emergence of FWGE as a subject of scientific inquiry, despite fermentation being an ancient food preservation technique. The modern perspective on FWGE is characterized by ongoing research into its potential applications, particularly supportive cancer care and immunomodulation (16, 19–21).

A notable feature of FWGE is its standardized production process, which involves extraction, fermentation, separation, drying, and granulation, resulting in a laboratory-standardized compound (16). This rigorous production approach distinguishes FWGE from many traditional fermented foods and aligns it more closely with modern nutraceutical development (16).

It is important to note that while FWGE has garnered attention for its potential health benefits, its status as a novel food is recognized by regulatory bodies. The European Food Safety Authority (EFSA) includes FWGE in its Novel Food Catalogue,^1^ highlighting that this food product was not consumed in the EU to a significant degree before May 15, 1997. Acknowledging its relatively recent introduction into the European market raises the need for careful evaluation of its safety and efficacy. This classification, as novel foods, highlights the evolving nature of food innovation and the regulatory challenges faced by novel fermented products.

Although a wide range of fermented cereal based products such as rice, oats, and barley have been studied for their nutritional properties, this review focuses exclusively on FWGE because of its unique profile and the availability of clinical evidence demonstrating potential anticancer effects. As research continues to explore the mechanisms and potential applications of FWGE, it will serve as an intriguing case study for the development of novel fermented foods. Ongoing scientific interest in FWGE exemplifies the potential of traditional fermentation processes to yield products with unique bioactive properties, warranting further investigation in the context of modern nutritional science and medicine.

By focusing on FWGE’s unique position as both a novel food and a subject of clinical investigation, this review aims to contribute to a broader understanding of how fermented foods can transition from traditional dietary components to scientifically validated health interventions.

This systematic review aimed to answer the following research questions:

- In which disease context, particularly cancer, has FWGE been investigated, and what outcomes have been reported?
- What is the evidence from preclinical and clinical studies on the efficacy, safety, and mechanisms of FWGE with a primary focus on its anticancer effects and secondary consideration of other health-related outcomes?

## Methods

### Systematic review of human studies

The systematic review was initially designed to evaluate the safety and functionality of novel fermented foods, with methodology and reporting structured according to the Preferred Reporting Items for Systematic Reviews and Meta-Analyses (PRISMA) guidelines to ensure both transparency and reproducibility. A comprehensive literature search was conducted, using a pre-defined search string that targeted publications related to the safety and functionality of novel fermented foods.

The initial search yielded 208 articles. Duplicate records were removed using CADIMA software (22), resulting in 143 unique articles for screening. Title and abstract screening were independently performed by two reviewers. A consistency check was performed to ensure the reliability of the selection process. Any disagreements between reviewers were resolved through discussion until a consensus was reached.

During the screening process, it became apparent that the available literature specifically addressing novel fermented foods, defined according to the EFSA Novel Food List, was limited. Only six articles met both the EFSA novel food criteria and the specific focus on FWGE. Given this scarcity and the specificity of the literature, the research team made a collective decision to narrow the focus of the systematic review to exclusively address FWGE.

### Literature search focusing on FWGE

A systematic literature search specifically targeting studies on FWGE was performed in three major electronic databases: PubMed, Scopus, and the Cochrane Library. The initial search included studies published between January 1, 1970, and August 31, 2023. To ensure that the review was up to date, a secondary search was conducted in January 2025 to capture relevant articles published between August 31, 2023, and December 31, 2024. Only articles published in English were considered eligible for inclusion.

For the search strategy, a generic search string originally developed by the Library of the University of Zurich (Alisa Berger) for the PIMENTO project was customized for FWGE in each database. In adapting the generic PIMENTO search string, rather than appending a functional search term, all references to other fermented commodities were removed and replaced with the term “fermented wheat germ extract” to focus the search exclusively on FWGE.

### Selection criteria

All human studies identified through the systematic search were screened for eligibility according to pre-specified inclusion and exclusion criteria. The review included both interventional studies (specifically, randomized controlled trials, non-randomized controlled trials, and uncontrolled intervention studies) and observational studies (including cohort, case–control, and cross-sectional studies) that investigated the health effects of FWGE in humans. Systematic reviews were also included during the initial screening to identify any potentially relevant primary studies that might have been missed. Animal and *in vitro* studies were excluded from this systematic review.

Studies were eligible if they included adult participants (age 18 years or older); studies focusing on adolescents or children (under 18 years of age) were excluded. Only studies reporting the health effects of FWGE attributable specifically to the fermentation process were included, while those examining effects related to non-fermentation components or unrelated dietary factors were excluded. For all studies meeting the criteria for population, intervention/exposure, and outcome, the presence and quality of a control group were assessed.

The primary clinical indications for FWGE, as reported by the included studies, were selected as the primary endpoints for this review. Where available, additional reported outcomes were extracted and summarized.

### Study selection and data extraction

The results of the literature search from each database were imported into CADIMA software for further management and analysis. Duplicate records were identified and removed using CADIMA’s automated tools, after which the remaining unique studies from all the databases were merged into a single dataset for screening. Title and abstract screening were performed independently by three reviewers. Studies not meeting the pre-specified selection criteria were excluded. To assess the reliability of the screening process, the consistency between reviewers was quantified using Cohen’s kappa coefficient, which yielded a value of 0.66, indicating “good” agreement. Any discrepancies in the inclusion or exclusion decisions were discussed and resolved collectively by the review team.

Full-text screening was conducted independently by all three reviewers for studies that passed the initial screening. Disagreements were resolved by discussion to achieve a consensus, resulting in the final list of studies to be included in the systematic review. The study selection and data extraction procedures were conducted according to the methodology described by Muka et al. (23). Two trials identified during the search (ChiCTR2000029726 and NCT02411565) were registered in trial databases but did not have peer-reviewed full texts available, and were excluded from the data extraction and synthesis. For data extraction, each article was reviewed by two reviewers working independently to extract all relevant information, including the study design, participant characteristics, intervention or exposure details, control group information, and reported outcomes. The extracted data were then compared and merged using reviewer pairs to ensure completeness and accuracy. The finalized data extraction files were used to build a review database for subsequent analysis. In addition, we performed backward citation tracking by screening the reference lists of included articles to identify further relevant studies. Any articles identified in this way were subjected to the same eligibility criteria and screening process as the database search results.

**Figure 1:**
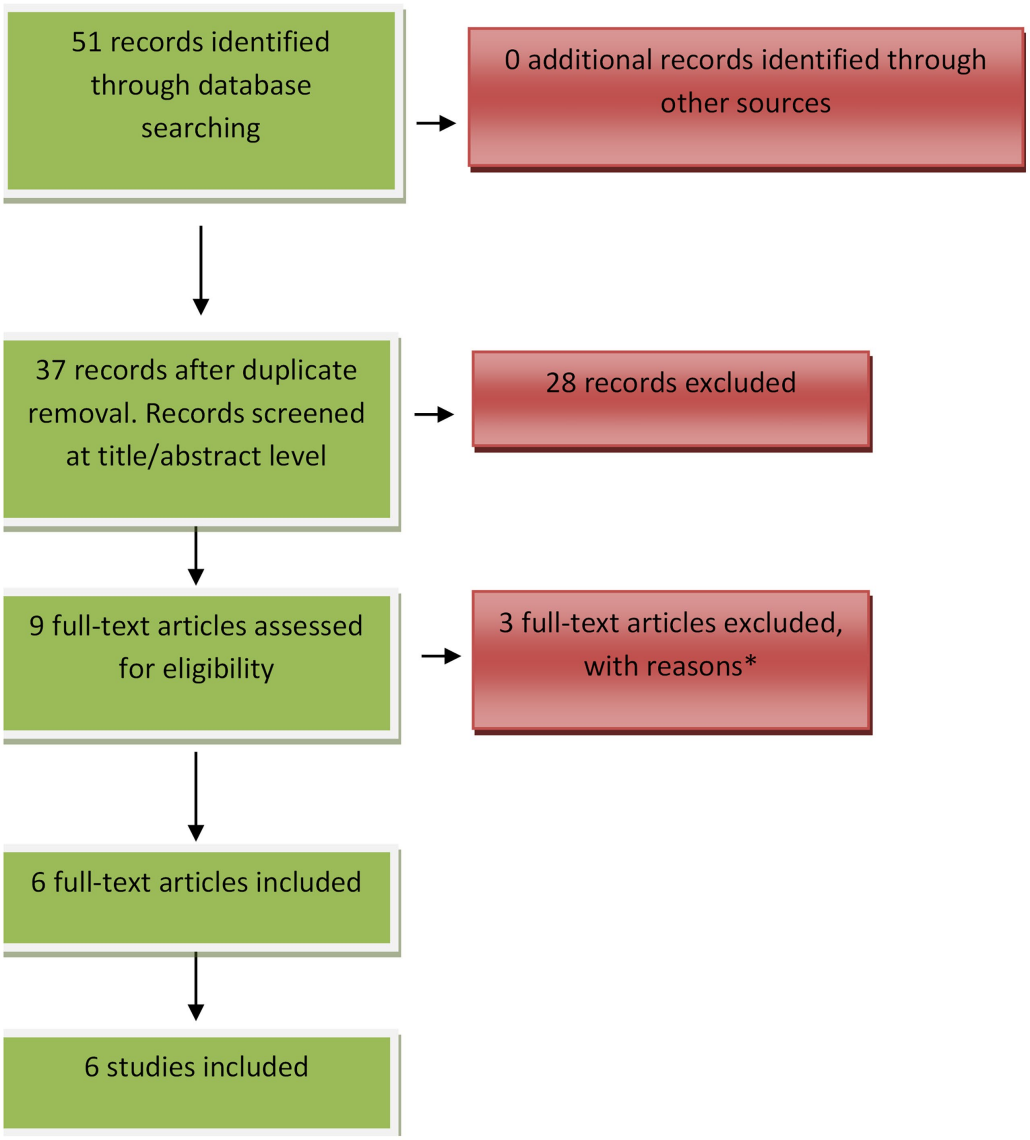
Flow diagram for systematic search. *Reasons for full-text exclusion: age limit (*n* = 1), full text not available (*n* = 2).

A flow diagram summarizing the study selection process and outcomes of the systematic search is provided in Figure 1.

### Data analysis

The methodology described by Muka et al. (23) was followed for the synthesis and analysis of data. After creating the review database, the team of reviewers synthesized the extracted data by defining key study characteristics, including type and amount of FWGE exposure, intervention details (such as duration and frequency), and reported outcomes. The findings from the included studies were summarized using narrative text, and the review process was documented step-by-step, with the number of studies at each stage presented in a flowchart.

Study characteristics, including population demographics, intervention protocols, exposure levels, control group descriptions, and outcomes, were systematically summarized and presented in a tabular format. Quantitative meta-analysis was not performed because of substantial heterogeneity and methodological differences among the included studies, such as variations in study design, assessed outcomes, and study populations.

### Bias and quality of study

The quality and risk of bias of the included studies (those with an appropriate control group) were independently assessed by two reviewers. Each reviewer completed the assessment using the relevant tools: the Newcastle–Ottawa Quality Assessment Scale for observational studies and the revised Cochrane risk-of-bias tool for randomized trials (ROB2) for intervention studies, selected according to study design. After independent assessments, the reviewers compared their results. Any discrepancies in the evaluation of the study quality or risk of bias were resolved through discussion until a consensus was reached. The detailed outcomes of the quality and bias assessments of the eligible studies are provided in Supplementary File.

### Non-systematic part of the review

To further characterize the functional aspects of FWGE and synthesize evidence regarding its bioavailability, mechanisms of action, and safety, we followed the guidelines and workflows provided by the PIMENTO COST Action CA20128 WG3 initiative, which are publicly available on the Open Science Framework (OSF) platform, as well as the EFSA framework, as published by Todorovic et al. (24). For product characterization, we extracted and summarized key information from the included studies, such as FWGE production methods, microbial strains used, principal bioactive constituents, and compositional variability. Any missing or incomplete information was supplemented through a secondary literature search that included evidence from animal and *in vitro* studies.

To synthesize supporting evidence regarding bioavailability and mechanisms of action, we adhered to EFSA guidance (section 5.2.3), prioritizing evidence from human, animal, and *in vitro* research. We specifically evaluated studies reporting the absorption, metabolism, and biological pathways through which FWGE may exert its effects.

Safety evaluation of FWGE (SP-29) was conducted by critically appraising safety-related data from selected human studies, which was complemented by a secondary screening of relevant animals and *in vitro* research to provide a comprehensive risk assessment. Safety outcomes, potential adverse effects, and impact of variability in FWGE production were documented and discussed.

### Summary of the systematic and non-systematic parts of the review

The analysis and synthesis of clinical evidence were conducted in accordance with the EFSA guidelines, specifically sections 5.2.1 and 5.2.2. This review focused on evaluating the relationship between FWGE consumption and its purported health effects. To assess the totality of scientific data, we considered the quality of FWGE characterization, strength and consistency of evidence for biological effects, extent to which a cause-and-effect relationship could be established from human clinical studies, and practicality of achieving effective FWGE consumption patterns in real-world settings. Although our research question was framed broadly to capture all disease areas where FWGE has been investigated, the literature was almost exclusively focused on cancer, with limited evidence in rheumatoid arthritis.

Our systematic search identified six clinical trials evaluating FWGE, all of which were published prior to 2010. No more recent clinical trials were retrieved. In addition, three animal studies and several cell culture studies were identified, which provide complementary insights into possible mechanisms of action.

## Results and discussion

### Biological plausibility

#### Characterization of FWGE

Wheat germ, comprising 2–3% of the total weight of the wheat kernel, is rich in phytochemicals, spermidine, benzoquinone molecules, B vitamins, polyunsaturated fatty acids, dietary fibers, minerals, *α*-tocopherol, and apigenin (25–27). It is also a valuable source of carbohydrates, primarily sucrose (ranging from approximately 56–78 g/100 g dry weight), and protein (11.2–30.0 g/100 g dry weight) (27–30). However, the use of wheat germ is limited by its poor stability and the presence of anti-nutritional factors such as raffinose, phytic acid, and wheat germ agglutinin (31–33). Wheat germ is commonly removed during the milling process due to its negative impact on the storage and processing quality of flour (34) Fermentation has proven to be an effective approach to address these challenges and improve the functionality of wheat germ for human consumption.

FWGE displays high antioxidant activity, likely attributable to an increase in free phenolic compounds and peptides generated during fermentation (35, 36). The bioactive compounds in FWGE and their functional properties are shown in Table 1. In addition, FWGE contains several other compounds of nutritional interest, including vitamins, especially vitamin E, and essential minerals, such as iron, manganese, copper, sodium, potassium, and sulfur. The concentrations of these nutrients were higher than those in unfermented wheat germ. Fermentation facilitates the breakdown of complex compounds, thereby enhancing the release and bioavailability of minerals for absorption and utilization in the body (30).

**Table 1:** Bioactive compounds in FWGE and their functional properties.

Bioactive compounds	Functional properties	References
2,6-Dimethoxy-1,4-benzoquinone (DMBQ)	Major anticancer activity Antiproliferative Redox modulation, antioxidant properties	(44, 72)
2-Methoxy-benzoquinone	Contributes to antiproliferative and antimetastatic effects Redox modulation, antioxidant properties	(16, 57, 72)
Peptides	Antioxidant Anticarcinogenic effects	(35)
Polyphenols	Antioxidant Anticarcinogenic effects	(35)

While native wheat germ contains physiologically inactive glycosylated quinones, fermentation, particularly via yeast glycosidases, releases bioactive quinones, such as benzoquinone, 2-methoxy benzoquinone, and 2,6-dimethoxy benzoquinone, which are present in FWGE. Quinones are cyclic organic compounds characterized by two carbonyl groups (C = O) incorporated within a conjugated ring structure (37). The content of released benzoquinones in FWGE is influenced by the fermentation conditions (38).

FWGE, standardized for methoxy-substituted benzoquinones, was registered in Hungary as a medical food in 2002. This patented product contains 2,6-dimethoxy-1,4-benzoquinone (DMBQ) and 2-methoxy-benzoquinone at concentrations of approximately 400 μg/g (0.04%) of the crude extract (26, 39). Although benzoquinones, peptides and phenolics are biologically active compounds in FWGE, a detailed characterization of the exact composition of the patented product is lacking. The nutrient profile of FWGE depends greatly on the fermentation conditions, such as the type of microorganism, pH, temperature, and fermentation time. Further research is necessary to optimize FWGE production for fortified health applications. The patented product FWGE was used in all six clinical trials analyzed in this study.

### Identification of pertinent human efficacy studies

The systematic search revealed six studies that investigated the effects of FWGE across various conditions (Table 2).

**Table 2:** Clinical applications of FWGE across cancer and autoimmune diseases: study designs, outcomes, and key findings.

Type of cancer or treated disease	Population size (n)	Study design	Intervention	Control	Outcomes	Key findings	References
Colorectal cancer	30	RCT	9 g FWGE daily + surgery/chemotherapy	Patients who received anticancer treatments (surgery, chemotherapy, radiotherapy) alone	Therapeutic benefit, Metastasis	No new metastases reported; Improved therapeutic benefit when combined with standard treatments	(40)
Malignant Skin Melanoma (with lymphatic metastasis)	58	Randomized	8.5 g FWGE daily+ Dacarbazine (DTIC) chemo	Patients receiving standard chemotherapy (DTIC) alone, without FWGE	Pogression-free survival (PFS) and overall survival (OS)	Significant improvement in PFS (55.8 vs. 29.9 months) and OS (66.2 vs. 44.7 months) (*p* = 0.0184)	(18)
Advanced head and neck cancer	60	Prospective	9 g FWGE daily (single/double dose)	Patients treated with conventional oncological therapy alone	Oxidative stress, Quality of Life	Improved QOL; No adverse effects; Stable oxidative stress markers	(41)
Colorectal cancer	176	Cohort Study	9 g FWGE daily + surgery/chemotherapy	Patients who received anticancer treatments (surgery, chemotherapy, radiotherapy) alone	Metastasis prevention, Survival Rates	Significantly fewer new recurrences; Lower mortality (*p* < 0.01); Delayed progression compared to controls	(57)
Castration-resistant prostate cancer (CRPC)	36	Pilot	8.5 g FWGE daily + GnRH therapy	Not Available	PSADT, Progression	23.1% showed significant increase in PSADT; Improved PSA levels; Delayed disease progression in one out of four patients	(42)
Rheumatoid arthritis	15	Open-label	8.5 g FWGE daily + DMARD/steroids;	Not Available	Disease Activity, Inflammatory Markers	Significant reduction in sedimentation rate; Improvement in Ritchie Index scores	(56)

chemo., chemotherapy; QOL, quality of life; PSADT, Prostate-specific antigen doubling time.

Jakab et al. (40) carried out a phase II clinical pilot study with FWGE to investigate its ability to provide additional therapeutic benefits following surgery or chemotherapy. The study was conducted between 1998 and 1999 on 18 patients who served as a control and 12 who received the MSC, either as adjuvant to chemotherapy or alone. Interim data, with a median follow-up of 9 months, indicated that no new metastases were observed in the MSC group, whereas four out of 18 patients developed new metastases in the control group. The results suggest that orally administered MSC may serve as promising supportive therapy in the treatment of colorectal cancer (31).

Demidov et al. (18) reported on the adjuvant use of FWGE in the treatment of patients suffering from malignant stage III skin melanoma with metastasis. In a randomized phase II clinical trial, patients receiving DTIC-based chemotherapy supplemented with FWGE showed a marked increase in both progression-free survival (PFS) and overall survival (OS) compared to the control group receiving the DTIC-based chemotherapy alone. Specifically, the mean PFS was 55.8 months in the FWGE group versus 29.9 months in the control group (*p* = 0.0137), and the mean OS was 66.2 months versus 44.7 months, respectively (*p* = 0.0298). Consequently, the authors recommend the integration of FWGE into adjuvant treatment protocols for patients with high-risk skin melanoma.

The impact of FWGE has also been evaluated other types of cancer. Oxidative stress in patients with advanced head and neck cancer undergoing conventional oncological treatments was notably reduced compared with those undergoing conventional treatment alone. The decrease in oxidative stress paralleled with a statistically significant improvement in the patients’ quality of life, as measured by the Spitzer QOL index (41).

A multicenter, open-label cohort study was undertaken to assess the effect of supplementing standard anticancer treatment with 9 g daily of FWGE daily on progression-free survival in colorectal cancer patients. A total of 176 colorectal cancer patients from three oncosurgical institutions in Hungary—Uzsoki Teaching Hospital (Budapest), the University of Szeged, and the University of Debrecen—were enrolled in the study. Sixty-six patients from three cancer centers who chose to receive MSC were given supplements for more than 6 months. These patients were compared with a control group of 104 patients who received only standard anticancer therapies. Although the time from diagnosis to the last visit was similar in both groups, the MSC group experienced significantly fewer instances of disease progression (new recurrences: 3.0% vs. 17.3%, *p* < 0.01; new metastases: 7.6% vs. 23.1%, *p* < 0.01; deaths: 12.1% vs. 31.7%, *p* < 0.01). Patients receiving MSC also showed notable improvements in progression-free survival (*p* = 0.0184) and overall survival (*p* = 0.0278). Thus, the study suggested that adding FWGE to standard anticancer regimens for at least 6 months may improve both overall and progression-free survival for colorectal cancer patients (39).

FWGE was also explored for its use in the treatment of castration resistant prostate cancer (CRPC). A pilot study (42) evaluated the efficacy of combined therapy using a GnRH analog and FWGE in 36 patients with CRPC. The primary endpoint was to assess if the combined therapy slowed disease progression, as measured by prostate specific antigen doubling time (PSADT). After at least 4 months of treatment, 11% of the patients experienced improved overall health and quality of life. Notably, 65.4% of patients (17 of 26) showed extended PSADT, with a statistically significant extension observed in six patients. These findings suggest that the addition of FWGE to conventional GnRH analog therapy may offer clinical benefits in some CRPC patients and potentially delay the need for chemotherapy (42).

Across the five cancer-related studies included in this review, FWGE demonstrated significant anticancer effects, such as prolonged progression-free and overall survival in melanoma and colorectal cancer, reduced oxidative stress and improved quality of life in patients with head and neck cancer, and delayed disease progression in prostate cancer.

### Quality and bias of human studies

This review has limited the evaluation of Q&B to the four studies with an adequate control group (20, 31, 39, 41). The absence of controls has a detrimental effect on the quality of articles. The Risk-of-Bias Tool for randomized trials (Rob 2) was used for randomized controlled trials (RCTs) (20, 31), whereas the Newcastle-Ottawa Quality Assessment Scale was used for cohort studies (39, 41).

As shown in Table 3, the overall risk of bias was judged to be low for one RCT (20) and one cohort study (39), medium for one cohort study (41), and high for one RCT (31).

**Table 3:** Q&B assessment of studies.

Article	Type of study	Method of Q&B analyses	D-1	D-2	D-3	D-4	D-5	Quality and risk of bias
Zhang et al. (31)	RCT	Rob 2	SC	HR	LR	HR	SC	High Risk
Demidov et al. (20)	RCT	Rob 2	LR	LR	LR	LR	LR	Low Risk

			Selection	Comparability	Outcome	
Zalatnai et al. (41)	Cohort	Newcastle-Ottawa QAS	2 stars	1 star	2 stars	Middle Quality (5 stars)
Tai et al. (39)	Cohort	Newcastle-Ottawa QAS	4 stars	1 star	2 stars	High Quality (7 stars)

D, Domain; D-1, Risk of bias arising from the randomization process; D-2, Risk of bias due to deviations from the intended interventions (effect of assignment to intervention); D-3, Missing outcome data; D-4, Risk of bias in measurement of the outcome; D-5, Risk of bias in selection of the reported result; LR, Low Risk; HR, High Risk; SC, Some Concerns.

The risk of bias for the study by Demidov et al. (20) RCT with a 7-years follow-up that showed improved survival in high-risk skin melanoma patients treated with FWGE, was judged as low for all domains, including the risk of bias resulting from the randomization process (D-1), deviations from the intended interventions (D-2), missing outcome data (D-3), measurement of the outcome (D-4), and selection of the reported result (D-5) (20).

In contrast, the RCT by Jakab et al. was rated as having a high risk of bias, particularly regarding deviations from the intended interventions (D-2) and measurement of the outcome (D-4). Some concerns are also raised regarding the randomization process (D-1) and the selection of the reported result (D-5) (31).

FWGE was found to have a supportive value in the treatment of colorectal cancer in the high-quality cohort study by Tai et al. (39) whose participants demographics were truly representative of the average colorectal cancer patients in the community. The non-exposed cohort was selected from the same community as the exposed cohort and a structured interview was used to demonstrate that the outcome of interest was not present at the start of the study. This study used a control group without MSC administration, and the outcomes were assessed independently. The follow-up of the cohorts was adequate, with only 3.4% of subjects lost.

Another cohort study (41) examined the impact of FWGE on the quality of life and oxidative stress in patients with advanced head and neck cancer, was scored as middle quality. The study population was representative of community cases of head and neck tumors, and the exposed and non-exposed cohorts were drawn from the same source. However, there was no description of how FWGE exposure was ascertained, nor was there any demonstration that the outcome of interest was present at the start of the study. The comparability of the cohorts was sufficiently good based on the design because the study controls were patients with head and neck cancer who had not been exposed to FWGE. The outcome was assessed using independent blind assessment and self-report. Five patients who did not survive the study period were excluded from data analysis. This small sample size is unlikely to introduce bias, but the lack of a specific follow-up duration is a weakness of this cohort study.

To summarize, the risk of bias and the quality of the studies varied across studies, and more rigorous RCTs and cohort studies are needed.

### Safety

Clinical investigations have indicated that the use of FWGE is safe, with no significant adverse effects recorded (39, 41). Despite an increasing number of preclinical and clinical studies, the exact active component of FWGE remains unidentified, making it challenging to fully explain the biochemical effects observed. Nevertheless, the use of FWGE has consistently been deemed safe, with laboratory tests revealing no adverse effects on renal or hepatic function (41).

One study specifically observed significant improvements in disease activity, assessed by the Ritchie Index, alongside better patient-reported outcomes, as measured by the Health Assessment Questionnaire, and reduced morning stiffness. Some participants were also able to decrease their steroid dosage, all without notable adverse effects (32).

The dosage regimen of FWGE (≥9 g/day) has been well tolerated by patients, with a favorable safety profile and minimal adverse events reported. These findings support the feasibility of sustained FWGE consumption as a component of supportive cancer care in clinical settings.

### Substantiation of causal relationship between consumption of fermented food and functional effect

FWGE demonstrates specific effects on cancer cell proliferation, apoptosis, and immunomodulation, distinct from general nutritional effects (33, 43). Multiple studies reported a dose–response relationship between FWGE concentration and the inhibition of cancer cell growth and induction of apoptosis (16, 44). FWGE has been shown to significantly inhibit tumor cell proliferation and promote apoptosis at doses achievable through oral supplementation (45).

Clinical trials evaluating FWGE have typically involved supplementation periods ranging from several months up to 1 year, which are sufficient to observe meaningful effects in the context of cancer treatment (20, 45). Positive outcomes are consistent across different research groups and clinical settings (20, 45). While the current body of evidence indicates a relationship between FWGE consumption and beneficial anticancer and immunomodulatory effects, it is important to note that most studies to date have been limited in size and methodological rigor. Although the results are promising, further large-scale, high-quality randomized controlled trials are needed to definitively substantiate a causal relationship.

### Characterization of the relationship between consumption of the FWGE and the functional effect

Clinical and preclinical evidence supports the anticancer efficacy of FWGE as an adjunct to conventional cancer therapy. The functional effects of FWGE have been investigated primarily in patients with colorectal cancer, melanoma, and advanced head and neck cancer, for whom FWGE supplementation may be particularly relevant. Notable studies include colorectal cancer patients undergoing surgery, chemotherapy, or radiotherapy (39); stage III melanoma patients receiving dacarbazine (DTIC) chemotherapy (20); and individuals with advanced head and neck cancer (46). These groups represent clinically meaningful populations with active malignancies and provide relevant evidence for the functional effects of FWGE in the target population.

The reported benefits of FWGE have been observed in controlled clinical settings, where the supplement is administered orally in combination with standard anticancer treatments. These studies included randomized controlled trials and multicenter cohort studies with rigorous clinical follow-up, ensuring that measured outcomes are applicable to real-world therapeutic practice. For example, long-term follow-up in the melanoma clinical trial demonstrated sustained benefits, with FWGE supplementation associated with significantly prolonged progression-free survival (55.8 vs. 29.9 months; *p* = 0.0137) and overall survival (66.2 vs. 44.7 months; *p* = 0.0298) compared to controls, and follow-up extending up to seven years (20). These findings indicate that the functional effect of FWGE can be maintained with continuous use over extended periods.

Clinical studies have consistently employed a daily oral dose of at least 9 g of FWGE administered for a minimum duration of 6 months. This regimen was effective in improving survival and quality of life outcomes in the study population. As a standardized dietary supplement, FWGE is not a component of the typical dietary intake; thus, the therapeutic doses used in clinical trials exceed the normal consumption of wheat germ. FWGE supplementation is intended as an adjunct intervention rather than a replacement for standard therapies.

Evidence from randomized controlled trials, such as the melanoma study (20) supports the efficacy and safety of FWGE supplementation in improving survival and quality of life. Additional cohort studies on colorectal cancer (39) further support these findings. However, the non-randomized design of some studies underscores the need for additional large-scale randomized controlled trials to confirm and generalize these results. Overall, the current evidence suggests a promising role for FWGE as part of oncological supportive care.

### Animal studies

In addition to its cytotoxic effects on malignant cell lines observed *in vitro*, FWGE has demonstrated *in vivo* tumor control in animal models of colon cancer and melanoma (43, 47, 48). For example, in F-344 rats, the administration of MSC (FWGE) along with a basal diet led to a substantial reduction in the incidence of aberrant crypt foci (ACF) per area, a decrease in the number of rats developing colon tumors, and a lower density of colon tumors. These findings highlighted that FWGE effectively suppressed the development of azoxymethane-induced ACF and colon neoplasia, as well as tumor multiplicity, particularly during the early stages of carcinogenesis (48).

FWGE has also been evaluated as monotherapy and in combination with chemotherapeutic agents in animal cancer models. In rats, co-administration of orally delivered FWGE with 5-fluorouracil (5-FU) injections resulted in greater inhibition of liver metastases arising from colon cancer than 5-FU alone (47). Levis et al. reported that FWGE was effective against non-Hodgkin lymphoma and inhibited proliferation of non-small cell lung cancer cells in both *in vitro* and *in vivo* models (49).

Greenberg and Frishman (50) assessed the anticancer efficacy of FWGE using a transplantable tumor model with human HT-29 cells in nude mice, following promising *in vitro* results. In this study, both the FWGE and 5-FU treatment groups exhibited significant tumor reduction compared to the controls, with the greatest effect observed in the high-dose FWGE group (*p* < 0.05). The tumor inhibition rates for the 5-FU, high-dose FWGE, and low-dose FWGE groups were 30, 42, and 41%, respectively, indicating that FWGE was at least as effective, if not more so, than 5-FU in reducing tumor growth. Despite these beneficial effects, some animal studies have reported less favorable outcomes when FWGE was used in combination with certain chemotherapeutic agents. For instance, Febles et al. (51), found that the addition of FWGE to dacarbazine, 5-FU, or doxorubicin in mouse cancer models did not enhance the cytostatic effects of these drugs.

**Figure 2:**
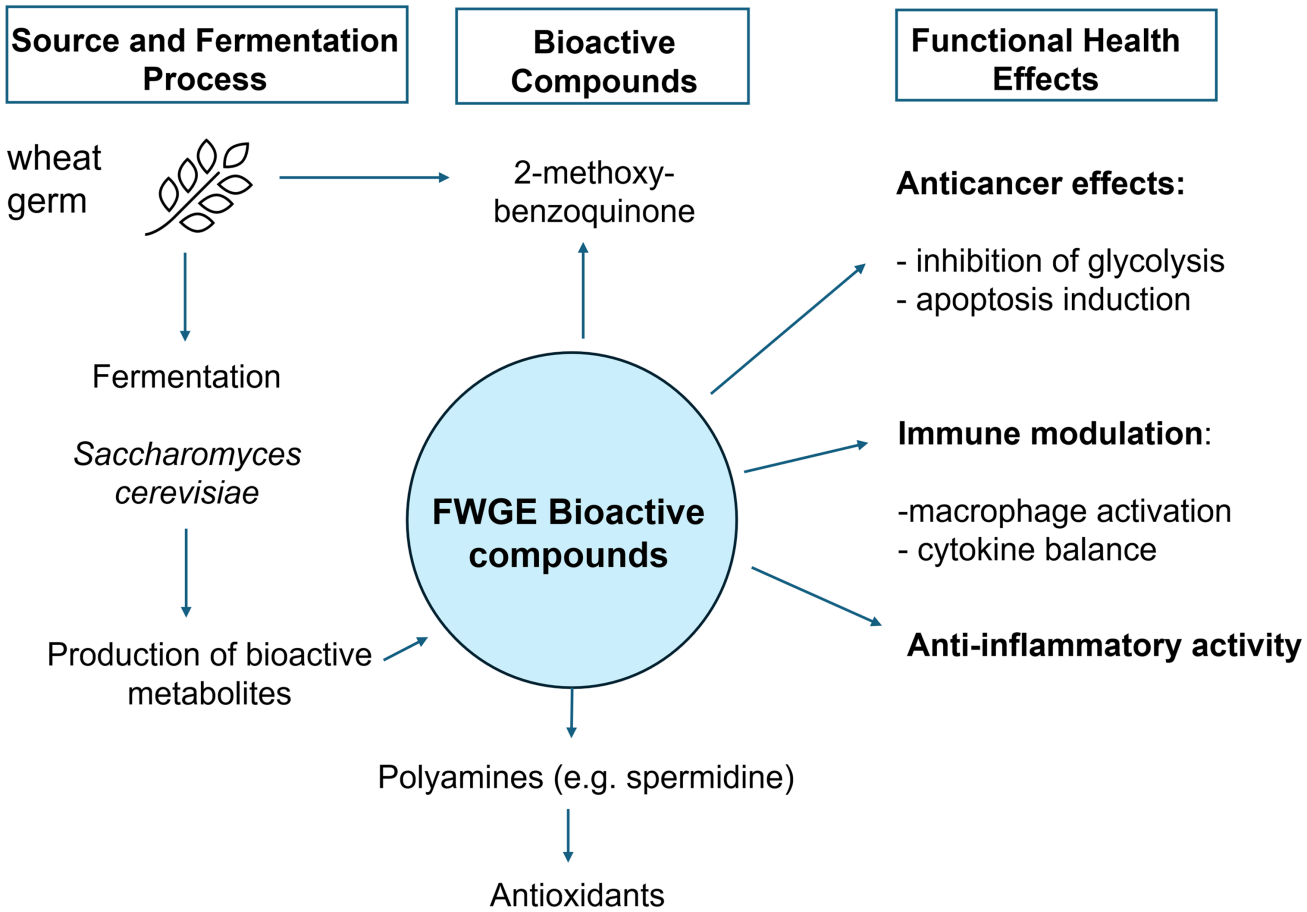
Functional and health-promoting roles of FWGE.

Beyond its anticancer and anti-inflammatory properties (Figure 2), FWGE has also been shown to impact cardiovascular and intestinal health in a limited number of animal studies (52, 53). In a rat model of hypertension and diet-induced obesity, Matucci et al. (52) demonstrated that FWGE treatment improved cardiac function, reduced collagen deposition in the ventricular myocardium by decreasing macrophage infiltration, reversed increased left ventricular stiffness in diseased hearts, and attenuated elevated plasma malondialdehyde concentrations. Additionally, FWGE reversed glucose intolerance, normalized systolic blood pressure, and reduced visceral fat accumulation in rats fed a high-fat/high-carbohydrate diet. These findings suggest a potential role of FWGE in alleviating cardiovascular complications associated with hypertension or diabetes.

### *In vitro* studies

FWGE (Avemar) is a dietary supplement with reported potential for cancer prevention and therapy (19, 46). Studies have demonstrated that wheat germ extract fermented with *Saccharomyces cerevisiae* possesses antioxidant, anti-inflammatory, and anticarcinogenic effects in a range of human cancer cell lines, including testicular, colon, melanoma, and leukemia cells (16, 54). The body of evidence supports the potential role of FWGE in cancer prevention and treatment (40, 55, 56).

One line of investigation focused on the antiproliferative and antimetabolic mechanisms of FWGE. Wang et al. (44) examined the effects of FWGE on nine cancer cell lines from various tissues, such as breast, pancreas, stomach, and colon. They reported that a 24-h treatment with 10 mg/mL FWGE resulted in a mean IC50 value indicative of an antiproliferative effect. FWGE exerted a cytotoxic effect in four cell lines [ASPC-1 and BxPC3 (pancreas), MDA-MB-231, and MDA-MB-468 (breast)], a cytostatic effect in another four [23,132/87 (stomach), BT-20 and MCF-7 (breast), and HT-29 (colon)], and growth retardation in HRT-18 (colon). The study also revealed that FWGE altered cell metabolism in 23,132/87 and HRT-18 cells, with a cytostatic effect in 23,132/87 cells linked to impaired glucose consumption and reduced lactic acid production. Additionally, FWGE-induced autophagic activity was observed in HRT-18 cells, as measured by LC3-II levels.

Studies have also explored the effects of FWGE fermented with lactic acid bacteria. Rizzello and colleagues reported that wheat germ fermented with lactic acid bacteria (*Lactobacillus plantarum*, *Lactobacillus brevis*, and *Lactobacillus rossiae*) suppressed the proliferation of colorectal cancer cell lines HT-29, HCT-8, and DLD-1 (57). Similarly, Zhang et al. showed that wheat germ extract fermented with *Lactobacillus plantarum* dy-1 inhibited HT-29 cell proliferation via induction of apoptosis (58).

The combination of FWGE with standard chemotherapeutics has been investigated in various cancer cell models (16). *In vitro*, FWGE has been shown to enhance the efficacy of chemotherapeutic agents such as tamoxifen in breast cancer cells (59), docetaxel in ovarian cancer cells (45, 60) and cisplatin in hepatocellular carcinoma cells (61). Studies have suggested that the addition of FWGE can allow for substantial dose reductions of chemotherapeutic agents, up to tenfold, while maintaining comparable antitumor effects (60, 61). Gatenby and Gillies (43) reported synergistic effects when FWGE was combined with 5-FU, oxaliplatin, or irinotecan across a diverse panel of human cancer cell lines including testicular cancer (H12.1, 2102EP, 1411HP, 1777NRpmet), colon cancer (HCT-8, HCT-15, HCT-116, HT-29, DLD-1, SW480, COLO205, COLO320DM), NSCLC (A549, A427, H322, H358), head and neck cancer (FADU, A253), cervical epidermoid carcinoma (A431), mammary adenocarcinoma (MCF-7, BT474), ovarian adenocarcinoma (A2780), gastric cancer (M2), anaplastic thyroid cancer (8505C, SW1736), papillary thyroid cancer (BCPAP), follicular thyroid cancer (FTC133), melanoma (518A2), hepatoma (HepG2), glioblastoma (U87MG), neuroblastoma (SHSY5Y, SIMA) cell lines. Hidvégi et al. (62) found that FWGE co-administered with cisplatin and docetaxel increased the cytotoxicity of these drugs in ovarian carcinoma cells (SKOV-3 and ES-2).

Additional *in vitro* studies highlighted FWGE’s antioxidant and anti-inflammatory activities. For example, in IPEC-J2 porcine intestinal epithelial cells, FWGE reduced oxidative stress and the inflammatory response induced by lipopolysaccharides (LPS) from *Salmonella typhimurium* and various *E. coli* strains (O55: B5, O111: B4, and O127: B8). FWGE treatment significantly decreased intracellular reactive oxygen species (ROS) and preserved cell layer integrity under LPS challenge, suggesting a protective effect against oxidative stress and barrier dysfunction (53).

### Mechanisms of action

Studies on the anticancer properties of FWGE have identified metabolic, antiproliferative, antimetastatic, and immunomodulatory effects as key mechanisms (16, 44, 47). These activities are attributed in part to the inhibition of cyclooxygenase isoforms and upregulation of endogenous antioxidants (52). While normal cells primarily direct glucose to undergo mitochondrial oxidative phosphorylation for ATP generation, cancer cells utilize glucose differently. FWGE targets the altered metabolism of cancer cells, which often rely on nonoxidative glucose utilization, resulting in increased lactic acid production by lactate dehydrogenase (LDH) (63).

FWGE reduces glucose uptake by directly inhibiting glucose activation as well as by inhibiting hexokinase, which catalyzes glucose phosphorylation. In addition, FWGE inhibits pentose cycle enzymes involved in direct glucose oxidation (glucose-6-phosphate dehydrogenase), and enzymes involved in non-oxidative glucose utilization for nucleic acid synthesis (transketolase). These inhibitions result in reduced glucose consumption by cancer cells, thereby slowing the progression of neoplastic diseases. FWGE also inhibits LDH, leading to decreased glycolytic flux and energy supply for tumor growth under both aerobic and anaerobic conditions. Furthermore, FWGE inhibits ribonucleotide reductase (RR) activity, a key enzyme in *de novo* DNA synthesis (33, 46). Inhibition of these pathways contributes to the antiproliferative capacity of FWGE. For instance, a study using the HT-29 human colon carcinoma cell line demonstrated that FWGE inhibited the activity of ribonucleotide reductase, further supporting its effect on DNA synthesis (40).

Induction of apoptosis is another important anticancer mechanism. Many anticancer drugs promote cell death via apoptosis, which is mediated by the activation of caspase-3 proteases. FWGE induces cleavage of poly (ADP-ribose) polymerase (PARP), a hallmark of apoptosis. FWGE has shown antitumor activity in numerous human cancer cell lines, including colon, testis, thyroid, ovarian, non-small cell lung cancer, breast, stomach, head and neck, hepatoma, glioblastoma, melanoma, cervix, and neuroblastoma (43). In many cases, the anticancer effect of FWGE is mediated by the induction of apoptosis via the caspase-PARP pathway (64, 65).

In addition, FWGE exerts immunomodulatory effects. It enhances natural killer (NK) cell activity by decreasing MHC-I molecule expression, increasing TNF secretion by macrophages, upregulating ICAM-1 expression in vascular endothelial cells, and boosting immune system activity. Enhanced NK cell activity is associated with decreased MHC-I antigen levels. TNF-*α* can kill tumor cells both directly (by inducing apoptosis and generating oxygen radicals) and indirectly (by inhibiting tumor angiogenesis and promoting other antitumor immune reactions). TNF-α also increases production of ICAM-1 molecules, which facilitate lymphocyte adhesion to target cells (66).

The composition of FWGE includes two quinones, 2-methoxy benzoquinone and 2,6-dimethoxy benzoquinone (DMBQ), present in wheat germ as glucosides and standardized to a 2,6-dimethoxy-p-benzoquinone content of 0.4 mg/g on a dry matter basis. These quinones are believed to be largely responsible for the anticancer activity of FWGE (16, 46). Furthermore, a protein sub-fraction known as “fermented wheat germ proteins” has demonstrated anticancer properties (67). Fermentation increases the levels of peptides and free phenolics while decreasing bound phenolics by altering protein-polyphenol interactions, and such changes may contribute to the biological activity of FWGE (36).

In addition to its anticancer properties, FWGE has attracted interest for its immunomodulatory functions via antioxidant and anti-inflammatory mechanisms (see the previous sections). However, the specific components responsible for these effects remain unclear. The constituents of FWGE, including benzoquinones and other plant flavonoids, may also confer cardioprotective effects. Benzoquinones are compounds with vitamin-like properties and antioxidant activity. For example, coenzyme Q10 (ubiquinone), a naturally occurring benzoquinone, helps protect cells during cardiac ischemia and reperfusion by supporting oxidative phosphorylation and stabilizing membranes (28). Epidemiological studies suggest that higher plasma levels or dietary intake of natural antioxidant vitamins may offer protection against cardiovascular diseases (19). Indicating that FWGE may have a potential role in cardiovascular health through oxidative stress regulation.

In summary, the mechanisms by which FWGE exerts its anticancer and therapeutic effects involve the disruption of tumor cell metabolism, induction of apoptosis, immunomodulatory actions, and antioxidant and anti-inflammatory activities. These actions are mediated by a range of molecules, including quinones, but may also involve bioactive peptides and phenolic compounds, the roles of which are still being elucidated.

### Current limitations in the research field and prospects

Despite promising evidence regarding the functionality and health benefits of FWGE, several important limitations must be acknowledged. These limitations include small sample sizes, short study durations, and methodological weaknesses commonly observed in clinical studies (20, 41, 42). Due to these limitations, caution is warranted when interpreting the generalizability of the findings regarding the functionality and anticancer effects of FWGE. Inconsistencies across studies, potentially due to differences in design, dosage, or study populations, further complicate interpretation. Additionally, there is currently no universal standard for FWGE production, indicating that different brands and formulations may vary considerably in quality and effectiveness (16). It is necessary to minimize variability within studies and enhance comparability between studies by optimizing characterization of the product, control, and reporting of products, data analysis. To enhance reproducibility, variability should be minimized (68). This lack of standardization makes it difficult for patients and clinicians to assess potency and safety. For future product development, it is essential to standardize FWGE production and characteristics, including optimizing the fermentation processes, ensuring consistent quality, and verifying both safety and efficacy. The production processes, pharmaceutical preparations, and applications of the most widely used FWGE product, Avemar, have been patented (WO2012018370A1). This product is fermented by *Saccharomyces cerevisiae*; however, the fermentation of the wheat germ can be implemented by other microorganisms such as *Lactobacillus* (69), *Aspergillus* (70), or mixed yeast-lactic acid bacteria culture (35). Not only is the choice of microorganism critical but also parameters such as starting material quality, preparation steps (e.g., cleaning, grinding), dilution ratio, fermentation time, temperature, and pH. A thorough understanding of product composition is needed to strengthen the scientific evidence for FWGE’s anticancer properties. This should include quantification of key bioactive compounds, particularly benzoquinones, as well as anti-nutritive compounds, phenolics, and peptides. While specific bioactive compounds such as 2-methoxy benzoquinone and 2,6-dimethoxy benzoquinone have been identified, a complete compositional characterization of FWGE has not been systematically reported in the literature, likely due to proprietary constraints. This is a critical gap, as benzoquinones are bioactive compounds with dose-dependent effects: at physiologically relevant concentrations they may contribute to anticancer activity, whereas at higher doses they can be toxic. Future studies are needed to provide a detailed biochemical profile of FWGE, including peptides, phenolic compounds, and other potential bioactive molecules, to better link its composition with its functional and clinical effects.

Diverse metabolic activities of microorganisms and variations in fermentation conditions can lead to products with widely differing properties. Although FWGE is primarily known for its anticancer effects, its bioactive profile suggests its potential benefits for a broader range of health conditions. For example, Szende et al. (32) investigated the effect of FWGE in patients with severe rheumatoid arthritis (RA) and found that continuous administration of FWGE as a supplement to standard therapies was beneficial. FWGE has also shown an antidepressant effect in a rat model, potentially through modulation of the gut-brain axis (71) In addition, FWGE reduced reactive oxygen species and lipid peroxidation during inflammation in rats (72). Studies in mice have demonstrated anti-aging properties, including improved organ indices, enhanced learning and memory, and reduced serum levels of total cholesterol, triglycerides, and glucose (73). Given that fermentation enhances the bioavailability of bioactive compounds in wheat germ, FWGE shows promise as a functional ingredient for managing various health conditions beyond adjunctive cancer therapy. Realizing this potential requires well-designed clinical trials.

## Conclusion

FWGE appears to be a promising functional extract for the adjunct treatment of cancer and other inflammatory diseases, with several studies suggesting a potential causal role in its therapeutic benefit. Among the six identified human studies, five focused on various cancer types, and one on rheumatoid arthritis. Four of these studies included proper controls; however, only half of them demonstrated high methodological quality. This highlights a key challenge: while some studies provide strong indications of causality, the overall body of evidence remains inconclusive due to limitations in study quality and sample size. Importantly, strong evidence for causality in specific, well-designed studies can coexist with broader, inconclusive literature. While the limited and dated nature of clinical evidence restricts definitive conclusions, this finding is itself significant, as it highlights the lack of follow-up studies despite initial promising results. The combination of preclinical, mechanistic, and clinical evidence assembled here allows us to contextualize FWGE’s potential effects, while also underlining the critical research gaps that remain. Therefore, more rigorous, high-quality clinical research is essential to confirm these findings and to support evidence-based recommendations.

## Data Availability

The original contributions presented in the study are included in the article/Supplementary material, further inquiries can be directed to the corresponding authors.

## Footnotes

^1^ https://ec.europa.eu/food/food-feed-portal/screen/novel-food-catalogue/search
