# Supplementary material for: A systematic review on the health effects of fermented wheat germ extract with emphasis on cancer
Source: Front Nutr. 2025 Nov 12;12:1677464. doi: 10.3389/fnut.2025.1677464 (PMC12649713; doi:10.3389/fnut.2025.1677464)
Supplement: Supplementary file 1 [file Table_1.docx]

Supplementary Table S1. Search strategy for bibliographic search of published human studies linked to fermented wheat germ extract.

| No. | Query |
| --- | --- |
| #1 | "fermented wheat germ extract"[tiab] |
| #2 | "Diet Surveys"[Mesh] OR "Cohort Studies"[Mesh] OR cohort*[Tiab] OR prospective[Tiab] OR longitudinal[Tiab] |
| #3 | Randomized Controlled Trial[Publication Type] OR Controlled Clinical Trial[Publication Type] OR Pragmatic Clinical Trial[Publication Type] OR Clinical Study[Publication Type] OR Adaptive Clinical Trial[Publication Type] OR Equivalence Trial[Publication Type] OR Clinical Trial[Publication Type] OR Clinical Trial, Phase I[Publication Type] OR Clinical Trial, Phase II[Publication Type] OR Clinical Trial, Phase III[Publication Type] OR Clinical Trial, Phase IV[Publication Type] OR Clinical Trial Protocol[Publication Type] OR multicenter study[Publication Type] OR "Clinical Studies as Topic"[Mesh] OR "Clinical Trials as Topic"[Mesh] OR "Clinical Trial Protocols as Topic"[Mesh] OR "Multicenter Studies as Topic"[Mesh] OR "Random Allocation"[Mesh] OR "Double-Blind Method"[Mesh] OR "Single-Blind Method"[Mesh] OR "Placebos"[Mesh:NoExp] OR "Control Groups"[Mesh] OR "Cross-Over Studies"[Mesh] OR random*[Title/Abstract] OR sham[Title/Abstract] OR placebo*[Title/Abstract] OR ((singl*[Title/Abstract] OR doubl*[Title/Abstract]) AND (blind*[Title/Abstract] OR dumm*[Title/Abstract] OR mask*[Title/Abstract])) OR ((tripl*[Title/Abstract] OR trebl*[Title/Abstract]) AND (blind*[Title/Abstract] OR dumm*[Title/Abstract] OR mask*[Title/Abstract])) OR "control study"[tiab:~3] OR "control studies"[tiab:~3] OR "control group"[tiab:~3] OR "control groups"[tiab:~3] OR "healthy volunteers"[tiab:~3] OR "control trial"[tiab:~3] OR "control trials"[tiab:~3] OR "controlled study"[tiab:~3] OR "controlled trial"[tiab:~3] OR "controlled studies"[tiab:~3] OR "controlled trials"[tiab:~3] OR "clinical study"[tiab:~3] OR "clinical studies"[tiab:~3] OR "clinical trial"[tiab:~3] OR "clinical trials"[tiab:~3] OR Nonrandom*[Title/Abstract] OR non random*[Title/Abstract] OR non-random*[Title/Abstract] OR quasi-random*[Title/Abstract] OR quasirandom*[Title/Abstract] OR "phase study"[tiab:~3] OR "phase studies"[tiab:~3] OR "phase trial"[tiab:~3] OR "phase trials"[tiab:~3] OR "crossover study"[tiab:~3] OR "crossover studies"[tiab:~3] OR "crossover trial"[tiab:~3] OR "crossover trials"[tiab:~3] OR "cross-over study"[tiab:~3] OR "cross-over studies"[tiab:~3] OR "cross-over trial"[tiab:~3] OR "cross-over trials"[tiab:~3] OR ((multicent*[tiab] OR multi-cent*[tiab] OR open label[tiab] OR open-label[tiab] OR equivalence[tiab] OR superiority[tiab] OR non-inferiority[tiab] OR noninferiority[tiab] OR quasiexperimental[tiab] OR quasi-experimental[tiab]) AND (study[tiab] OR studies[tiab] OR trial*[tiab])) OR allocated[tiab] OR pragmatic study[tiab] OR pragmatic studies[tiab] OR pragmatic trial*[tiab] OR practical trial*[tiab] |
| #4 | "Epidemiologic Methods"[Mesh:NoExp] OR "Epidemiologic Studies"[Mesh] OR "Observational Studies as Topic"[Mesh] OR "Clinical Studies as Topic"[Mesh] OR "Single-Case Studies as Topic"[Mesh] OR "Organizational Case Studies"[Mesh] OR observational study[Publication Type] OR validation study[Publication Type] OR clinical study[Publication Type] OR case reports[Publication Type] OR "observational study"[tiab:~3] OR "observational studies"[tiab:~3] OR "observational design"[tiab:~3] OR "observational analysis"[tiab:~3] OR "observational analyses"[tiab:~3] OR ((cohort*[tiab] OR prospective[tiab] OR follow-up[tiab] OR longitudinal[tiab] OR long-term[tiab] OR retrospective[tiab]) AND (study[tiab] OR studies[tiab] OR design[tiab] OR analysis[tiab] OR analyses[tiab] OR data[tiab] OR review[tiab])) OR case control*[tiab] OR case comparison*[tiab] OR case-referent[tiab] OR "population study"[tiab:~3] OR "population studies"[tiab:~3] OR "population analysis"[tiab:~3] OR "population analyses"[tiab:~3] OR "descriptive study"[tiab:~3] OR "descriptive studies"[tiab:~3] OR "descriptive design"[tiab:~3] OR "descriptive analysis"[tiab:~3] OR "descriptive analyses"[tiab:~3] OR "multidimensional study"[tiab:~3] OR "multidimensional studies"[tiab:~3] OR "multidimensional design"[tiab:~3] OR "multidimensional analysis"[tiab:~3] OR "multidimensional analyses"[tiab:~3] OR "cross-sectional study"[tiab:~3] OR "cross-sectional studies"[tiab:~3] OR "cross-sectional design"[tiab:~3] OR "cross-sectional analysis"[tiab:~3] OR "cross-sectional analyses"[tiab:~3] OR "cross-sectional research"[tiab:~3] OR "cross-sectional survey"[tiab:~3] OR "cross-sectional findings"[tiab:~3] OR natural experiment*[tiab] OR quasi experiment*[tiab] OR "nonexperimental study"[tiab:~3] OR "nonexperimental studies"[tiab:~3] OR "nonexperimental design"[tiab:~3] OR "nonexperimental analysis"[tiab:~3] OR "nonexperimental analyses"[tiab:~3] OR "prevalence study"[tiab:~3] OR "prevalence studies"[tiab:~3] OR "prevalence analysis"[tiab:~3] OR "prevalence analyses"[tiab:~3] OR case series[tiab] OR "case report"[tiab:~3] OR "case reports"[tiab:~3] OR "case study"[tiab:~3] OR "case studies"[tiab:~3] OR "case histories"[tiab:~3] |
| #5 | "systematic review" |
| #6 | #2 OR #3 OR #4 OR #5 |
| #7 | #1 AND #6 |
| #8 | #7 NOT (("Child"[Mesh] OR "Infant"[Mesh] OR "Adolescent"[Mesh]) NOT "Adult"[Mesh]) |
| #9 | #8 NOT (("Animals"[Mesh] OR "Animal Experimentation"[Mesh] OR "Models, Animal"[Mesh] OR "Vertebrates"[Mesh]) NOT ("Humans"[Mesh] OR "Human Experimentation"[Mesh])) |
| #10 | #9 NOT ("Breast Feeding"[Majr] OR "Milk, Human"[Majr]) |
| #11 | #10 AND (English[Filter]) |
| #12 | #11 AND (("1970/01/01"[Date - Publication] : "2023/08/31"[Date - Publication])) |
