# Supplementary material for: A systematic review on the health effects of fermented wheat germ extract with emphasis on cancer
Source: Front Nutr. 2025 Nov 12;12:1677464. doi: 10.3389/fnut.2025.1677464 (PMC12649713; doi:10.3389/fnut.2025.1677464)
Supplement: Supplementary file 2 [file Table_2.docx]

Revised Cochrane risk-of-bias tool for randomized trials (RoB 2)

TEMPLATE FOR COMPLETION

Edited by Julian PT Higgins, Jelena Savović, Matthew J Page, Jonathan AC Sterne
on behalf of the RoB2 Development Group

**Version of 22 August 2019**

The development of the RoB 2 tool was supported by the MRC Network of Hubs for Trials Methodology Research (MR/L004933/2- N61), with the support of the host MRC ConDuCT-II Hub (Collaboration and innovation for Difficult and Complex randomised controlled Trials In Invasive procedures - MR/K025643/1), by MRC research grant MR/M025209/1, and by a grant from The Cochrane Collaboration.


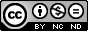


This work is licensed under a [Creative Commons Attribution-NonCommercial-NoDerivatives 4.0 International License](http://creativecommons.org/licenses/by-nc-nd/4.0/).

| **Study details**   \| **Reference** \| Demidov, L. V., Manziuk, L. V., Kharkevitch, G. Y., Pirogova, N. A., & Artamonova, E. V. (2008). Adjuvant fermented wheat germ extract (Avemar™) nutraceutical improves survival of high-risk skin melanoma patients: A randomized, pilot, phase ii clinical study with a 7-year follow-up. Cancer biotherapy & radiopharmaceuticals, 23(4), 477-482. \| \| --- \| --- \|   **Study design**   \| X \| Individually-randomized parallel-group trial \| \| --- \| --- \| \| □ \| Cluster-randomized parallel-group trial \| \| □ \| Individually randomized cross-over (or other matched) trial \|   **For the purposes of this assessment, the interventions being compared are defined as**   \| Experimental: \| the efficacy of dacarbazine (DTIC)+ fermented wheat germ extract (FWGE) \| Comparator: \| DTIC \| \| --- \| --- \| --- \| --- \|  \| **Specify which outcome is being assessed for risk of bias** \| progression-free (PFS) and overall survival (OS) \| \| --- \| --- \|  \| **Specify the numerical result being assessed.** In case of multiple alternative analyses being presented, specify the numeric result (e.g. RR = 1.52 (95% CI 0.83 to 2.77) and/or a reference (e.g. to a table, figure or paragraph) that uniquely defines the result being assessed. \| PFS mean values were: 55.8 (FWGE) versus 29.9 months (control) (p = 0.0137). OS mean values were: 66.2 (FWGE) versus 44.7 months (control) (p = 0.0298). \| \| --- \| --- \|   **Is the review team’s aim for this result…?**   \| □ \| to assess the effect of *assignment to intervention* (the ‘intention-to-treat’ effect) \| \| --- \| --- \| \| □ \| to assess the effect of *adhering to intervention* (the ‘per-protocol’ effect) \|   **If the aim is to assess the effect of *adhering to intervention***, select the deviations from intended intervention that should be addressed (at least one must be checked):  □ occurrence of non-protocol interventions  □ failures in implementing the intervention that could have affected the outcome  □ non-adherence to their assigned intervention by trial participants  **Which of the following sources were obtained to help inform the risk-of-bias assessment? (tick as many as apply)**  ■ Journal article(s) with results of the trial  □ Trial protocol  □ Statistical analysis plan (SAP)  □ Non-commercial trial registry record (e.g. ClinicalTrials.gov record)  □ Company-owned trial registry record (e.g. GSK Clinical Study Register record)  □ “Grey literature” (e.g. unpublished thesis)  □ Conference abstract(s) about the trial  □ Regulatory document (e.g. Clinical Study Report, Drug Approval Package)  □ Research ethics application  □ Grant database summary (e.g. NIH RePORTER or Research Councils UK Gateway to Research)  □ Personal communication with trialist  □ Personal communication with the sponsor |
| --- | --- | --- | --- | --- | --- | --- | --- | --- | --- | --- | --- | --- | --- | --- | --- | --- | --- | --- | --- | --- |

## Risk of bias assessment

Responses underlined in green are potential markers for low risk of bias, and responses in red are potential markers for a risk of bias. Where questions relate only to sign posts to other questions, no formatting is used.

**Domain 1: Risk of bias arising from the randomization process**

| **Signalling questions** | **Comments** | **Response options** |
| --- | --- | --- |
| **1.1 Was the allocation sequence random?** |  | Y / PY / PN / N / NI |
| **1.2 Was the allocation sequence concealed until participants were enrolled and assigned to interventions?** |  | Y / PY / PN / N / NI |
| **1.3 Did baseline differences between intervention groups suggest a problem with the randomization process?** |  | Y / PY / PN / N / NI |
| **Risk-of-bias judgement** |  | Low / High / Some concerns |
| Optional: What is the predicted direction of bias arising from the randomization process? |  | NA / Favours experimental / Favours comparator / Towards null /Away from null / Unpredictable |

Domain 2: Risk of bias due to deviations from the intended interventions (*effect of assignment to intervention*)

| **Signalling questions** | **Comments** | **Response options** |
| --- | --- | --- |
| **2.1. Were participants aware of their assigned intervention during the trial?** | Randomized study, | Y / PY / PN / N / NI |
| **2.2. Were carers and people delivering the interventions aware of participants' assigned intervention during the trial?** |  | Y / PY / PN / N / NI |
| **2.3. If Y/PY/NI to 2.1 or 2.2: Were there deviations from the intended intervention that arose because of the trial context?** |  | NA / Y / PY / PN / N / NI |
| **2.4 If Y/PY to 2.3: Were these deviations likely to have affected the outcome?** |  | NA / Y / PY / PN / N / NI |
| **2.5. If Y/PY/NI to 2.4: Were these deviations from intended intervention balanced between groups?** |  | NA / Y / PY / PN / N / NI |
| **2.6 Was an appropriate analysis used to estimate the effect of assignment to intervention?** |  | Y / PY / PN / N / NI |
| **2.7 If N/PN/NI to 2.6: Was there potential for a substantial impact (on the result) of the failure to analyse participants in the group to which they were randomized?** |  | NA / Y / PY / PN / N / NI |
| **Risk-of-bias judgement** |  | Low / High / Some concerns |
| Optional: What is the predicted direction of bias due to deviations from intended interventions? |  | NA / Favours experimental / Favours comparator / Towards null /Away from null / Unpredictable |

Domain 2: Risk of bias due to deviations from the intended interventions (*effect of adhering to intervention*)

| **Signalling questions** | **Comments** | **Response options** |
| --- | --- | --- |
| **2.1. Were participants aware of their assigned intervention during the trial?** |  | Y / PY / PN / N / NI |
| **2.2. Were carers and people delivering the interventions aware of participants' assigned intervention during the trial?** |  | Y / PY / PN / N / NI |
| **2.3. [If applicable:] If Y/PY/NI to 2.1 or 2.2: Were important non-protocol interventions balanced across intervention groups?** |  | NA / Y / PY / PN / N / NI |
| **2.4. [If applicable:] Were there failures in implementing the intervention that could have affected the outcome?** |  | NA / Y / PY / PN / N / NI |
| **2.5. [If applicable:] Was there non-adherence to the assigned intervention regimen that could have affected participants’ outcomes?** |  | NA / Y / PY / PN / N / NI |
| **2.6. If N/PN/NI to 2.3, or Y/PY/NI to 2.4 or 2.5: Was an appropriate analysis used to estimate the effect of adhering to the intervention?** |  | NA / Y / PY / PN / N / NI |
| **Risk-of-bias judgement** |  | Low / High / Some concerns |
| Optional: What is the predicted direction of bias due to deviations from intended interventions? |  | NA / Favours experimental / Favours comparator / Towards null /Away from null / Unpredictable |

Domain 3: Missing outcome data

| **Signalling questions** | **Comments** | **Response options** |
| --- | --- | --- |
| **3.1 Were data for this outcome available for all, or nearly all, participants randomized?** |  | Y / PY / PN / N / NI |
| **3.2 If N/PN/NI to 3.1: Is there evidence that the result was not biased by missing outcome data?** |  | NA / Y / PY / PN / N |
| **3.3 If N/PN to 3.2: Could missingness in the outcome depend on its true value?** |  | NA / Y / PY / PN / N / NI |
| **3.4 If Y/PY/NI to 3.3: Is it likely that missingness in the outcome depended on its true value?** |  | NA / Y / PY / PN / N / NI |
| **Risk-of-bias judgement** |  | Low / High / Some concerns |
| Optional: What is the predicted direction of bias due to missing outcome data? |  | NA / Favours experimental / Favours comparator / Towards null /Away from null / Unpredictable |

Domain 4: Risk of bias in measurement of the outcome

| **Signalling questions** | **Comments** | **Response options** |
| --- | --- | --- |
| **4.1 Was the method of measuring the outcome inappropriate?** |  | Y / PY / PN / N / NI |
| **4.2 Could measurement or ascertainment of the outcome have differed between intervention groups?** |  | Y / PY / PN / N / NI |
| **4.3 If N/PN/NI to 4.1 and 4.2: Were outcome assessors aware of the intervention received by study participants?** |  | NA / Y / PY / PN / N / NI |
| **4.4 If Y/PY/NI to 4.3: Could assessment of the outcome have been influenced by knowledge of intervention received?** |  | NA / Y / PY / PN / N / NI |
| **4.5 If Y/PY/NI to 4.4:** **Is it likely that assessment of the outcome was influenced by knowledge of intervention received?** |  | NA / Y / PY / PN / N / NI |
| **Risk-of-bias judgement** |  | Low / High / Some concerns |
| Optional: What is the predicted direction of bias in measurement of the outcome? |  | NA / Favours experimental / Favours comparator / Towards null /Away from null / Unpredictable |

Domain 5: Risk of bias in selection of the reported result

| **Signalling questions** | **Comments** | **Response options** |
| --- | --- | --- |
| **5.1 Were the data that produced this result analysed in accordance with a pre-specified analysis plan that was finalized before unblinded outcome data were available for analysis?** |  | Y / PY / PN / N / NI |
| **Is the numerical result being assessed likely to have been selected, on the basis of the results, from...** |  |  |
| **5.2. ... multiple eligible outcome measurements (e.g. scales, definitions, time points) within the outcome domain?** |  | Y / PY / PN / N / NI |
| **5.3 ... multiple eligible analyses of the data?** |  | Y / PY / PN / N / NI |
| **Risk-of-bias judgement** |  | Low / High / Some concerns |
| Optional: What is the predicted direction of bias due to selection of the reported result? |  | NA / Favours experimental / Favours comparator / Towards null /Away from null / Unpredictable |

Overall risk of bias

| **Risk-of-bias judgement** |  | Low / High / Some concerns |
| --- | --- | --- |
| Optional: What is the overall predicted direction of bias for this outcome? |  | NA / Favours experimental / Favours comparator / Towards null /Away from null / Unpredictable |


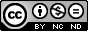


This work is licensed under a [Creative Commons Attribution-NonCommercial-NoDerivatives 4.0 International License](http://creativecommons.org/licenses/by-nc-nd/4.0/).

Revised Cochrane risk-of-bias tool for randomized trials (RoB 2)

TEMPLATE FOR COMPLETION

Edited by Julian PT Higgins, Jelena Savović, Matthew J Page, Jonathan AC Sterne
on behalf of the RoB2 Development Group

**Version of 22 August 2019**

The development of the RoB 2 tool was supported by the MRC Network of Hubs for Trials Methodology Research (MR/L004933/2- N61), with the support of the host MRC ConDuCT-II Hub (Collaboration and innovation for Difficult and Complex randomised controlled Trials In Invasive procedures - MR/K025643/1), by MRC research grant MR/M025209/1, and by a grant from The Cochrane Collaboration.


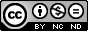


This work is licensed under a [Creative Commons Attribution-NonCommercial-NoDerivatives 4.0 International License](http://creativecommons.org/licenses/by-nc-nd/4.0/).

| **Study details**   \| **Reference** \|  \| \| --- \| --- \|   **Study design**   \| X \| **Individually-randomized parallel-group trial** \| \| --- \| --- \| \| □ \| Cluster-randomized parallel-group trial \| \| □ \| Individually randomized cross-over (or other matched) trial \|   **For the purposes of this assessment, the interventions being compared are defined as**   \| Experimental: \| The MSC group (12 patients) received MSC alone or plus adjuvant chemotherapy. \| Comparator: \| The control group (18 patients) received no other therapy or adjuvant chemotherapy alone. \| \| --- \| --- \| --- \| --- \|  \| **Specify which outcome is being assessed for risk of bias** \| Orally administered MSC is a potent candidate to be regarded as a supportive therapy to surgery or plus chemotherapy for colorectal cancer patients.  Appearence of new metastases \| \| --- \| --- \|  \| **Specify the numerical result being assessed.** In case of multiple alternative analyses being presented, specify the numeric result (e.g. RR = 1.52 (95% CI 0.83 to 2.77) and/or a reference (e.g. to a table, figure or paragraph) that uniquely defines the result being assessed. \| No new metastases have developed in the MSC group. Three patients of the control group developed metastases \| \| --- \| --- \|   **Is the review team’s aim for this result…?**   \| □ \| **to assess the effect of *assignment to intervention* (the ‘intention-to-treat’ effect)** \| \| --- \| --- \| \| □ \| to assess the effect of *adhering to intervention* (the ‘per-protocol’ effect) \|   **If the aim is to assess the effect of *adhering to intervention***, select the deviations from intended intervention that should be addressed (at least one must be checked):  □ occurrence of non-protocol interventions  □ failures in implementing the intervention that could have affected the outcome  □ non-adherence to their assigned intervention by trial participants  **Which of the following sources were obtained to help inform the risk-of-bias assessment? (tick as many as apply)**  □ Journal article(s) with results of the trial  □ Trial protocol  □ Statistical analysis plan (SAP)  □ Non-commercial trial registry record (e.g. ClinicalTrials.gov record)  □ Company-owned trial registry record (e.g. GSK Clinical Study Register record)  □ “Grey literature” (e.g. unpublished thesis)  □ Conference abstract(s) about the trial  □ Regulatory document (e.g. Clinical Study Report, Drug Approval Package)  □ Research ethics application  □ Grant database summary (e.g. NIH RePORTER or Research Councils UK Gateway to Research)  □ Personal communication with trialist  □ Personal communication with the sponsor |
| --- | --- | --- | --- | --- | --- | --- | --- | --- | --- | --- | --- | --- | --- | --- | --- | --- | --- | --- | --- | --- |

## Risk of bias assessment

Responses underlined in green are potential markers for low risk of bias, and responses in red are potential markers for a risk of bias. Where questions relate only to sign posts to other questions, no formatting is used.

**Domain 1: Risk of bias arising from the randomization process**

| **Signalling questions** | **Comments** | **Response options** |
| --- | --- | --- |
| **1.1 Was the allocation sequence random?** | 1.1. The methodology section mentions that patients in the MSC group were randomly assigned to receive MSC once or twice, but no information is provided regarding the randomization process for the study groups at baseline. Therefore, it can be inferred that randomization was applied only for the MSC dosage, but the process of assigning patients to groups is not explained.  1.2. The methodology section does not provide information about whether there was a concealed allocation process when assigning interventions after the participants were enrolled. Due to limited information on the randomization process, it is not possible to assess whether the group assignment was concealed before enrollment. | Y / PY / PN / N / NI |
| **1.2 Was the allocation sequence concealed until participants were enrolled and assigned to interventions?** |  | Y / PY / PN / N / NI |
| **1.3 Did baseline differences between intervention groups suggest a problem with the randomization process?** | Although the baseline characteristics are mentioned in the study, there is no clear evidence of a significant imbalance between the two groups. Some patients in the MSC group had metastases, but this does not appear to be an imbalance that would suggest issues with randomization. Based on the available information, there doesn't seem to be any evidence that the baseline differences between the two groups stemmed from the randomization process. However, this is not explicitly stated in the study. Therefore, it would be categorized as PN (Probably No) or NI (No Information). | Y / PY / PN / N / NI |
| **Risk-of-bias judgement** | In 1.3. whether the answer is PN or NI, it does not change the result. In both cases, according to the algorithm, the result is 'some concerns'. | Low / High / Some concerns |
| Optional: What is the predicted direction of bias arising from the randomization process? |  | NA / Favours experimental / Favours comparator / Towards null /Away from null / Unpredictable |

Domain 2: Risk of bias due to deviations from the intended interventions (*effect of assignment to intervention*)

| **Signalling questions** | **Comments** | **Response options** |
| --- | --- | --- |
| **2.1. Were participants aware of their assigned intervention during the trial?** | 2.1. The methodology section states that MSC was taken orally and participants were randomized to receive it once or twice daily. In this case, participants in the MSC group may be aware that they were receiving MSC, while those in the control group may be aware that they did not receive any additional treatment. Additionally, since no placebo was used in the study, it is likely that participants could have known which group they were in. Based on this information, the answer "probably yes" seems appropriate.  2.2. The methodology section does not provide information on whether the healthcare workers (caregivers or treatment providers) knew which treatment was given to patients in each group. However, because MSC was administered orally and in a specific dosage, it is likely that caregivers and treatment providers would have noticed that participants in the treatment group were receiving MSC.  Therefore, the most appropriate answer would be "probably yes". | Y / PY / PN / N / NI |
| **2.2. Were carers and people delivering the interventions aware of participants' assigned intervention during the trial?** |  | Y / PY / PN / N / NI |
| **2.3. If Y/PY/NI to 2.1 or 2.2: Were there deviations from the intended intervention that arose because of the trial context?**  **Içerikten kaynaklı sapmalar oldu mu?** | The study methodology provides a clear protocol for the dosage and duration of MSC administration. There is no information indicating that any changes were made to the treatment plan or that deviations from the intervention occurred due to the study context. Furthermore, based on the results, it appears that all patients in the MSC group received the treatment regularly, which suggests adherence to the protocol. Therefore, the answer "probably no" seems appropriate. On the other hand, since there is no explicit information regarding any deviations from the intervention in the study, the "not specified" option would also be suitable. This situation prevents us from definitively assessing whether there were any deviations arising from the study context. | NA / Y / PY / PN / N / NI |
| **2.4 If Y/PY to 2.3: Were these deviations likely to have affected the outcome?** | Not necessary | NA / Y / PY / PN / N / NI |
| **2.5. If Y/PY/NI to 2.4: Were these deviations from intended intervention balanced between groups?** | Not necessary | NA / Y / PY / PN / N / NI |
| **2.6 Was an appropriate analysis used to estimate the effect of assignment to intervention?** | The methodology section does not provide any information about the specific analysis method or statistical model used to assess the effects between the groups. However, it is mentioned that randomization was performed and treatment differences between the groups were examined. In this context, the term "appropriate analysis" refers to the need for statistically correct comparison of effects between the groups. Since there is no clear information on whether the study used an appropriate analysis to compare effects between the groups, the answer "no information" may be more appropriate. | Y / PY / PN / N / NI |
| **2.7 If N/PN/NI to 2.6: Was there potential for a substantial impact (on the result) of the failure to analyse participants in the group to which they were randomized?** | n terms of the study content:  The article's methodology and results sections do not provide clear information that participants were analyzed according to the groups they were randomized into. However, the results for the MSC group and the control group are reported separately, which suggests that the data may have been analyzed based on randomization. Considering that the authors presented the findings by group, the "probably no" option could also be considered, but it is unclear whether there were deviations from the data. Since it is not explicitly stated whether the analysis was performed based on the randomized groups, the "no information" option seems more appropriate in this case. | NA / Y / PY / PN / N / NI |
| **Risk-of-bias judgement** | Section 1 up to 2.3: some concerns Section 2, including 2.6 and 2.7: High risk  Overall: according to the algorithm, the result is ‘High risk’ | Low / High / Some concerns |
| Optional: What is the predicted direction of bias due to deviations from intended interventions? |  | NA / Favours experimental / Favours comparator / Towards null /Away from null / Unpredictable |

Domain 2: Risk of bias due to deviations from the intended interventions (*effect of adhering to intervention*)

| **Signalling questions** | **Comments** | **Response options** |
| --- | --- | --- |
| **2.1. Were participants aware of their assigned intervention during the trial?** |  | Y / PY / PN / N / NI |
| **2.2. Were carers and people delivering the interventions aware of participants' assigned intervention during the trial?** |  | Y / PY / PN / N / NI |
| **2.3. [If applicable:] If Y/PY/NI to 2.1 or 2.2: Were important non-protocol interventions balanced across intervention groups?** |  | NA / Y / PY / PN / N / NI |
| **2.4. [If applicable:] Were there failures in implementing the intervention that could have affected the outcome?** |  | NA / Y / PY / PN / N / NI |
| **2.5. [If applicable:] Was there non-adherence to the assigned intervention regimen that could have affected participants’ outcomes?** |  | NA / Y / PY / PN / N / NI |
| **2.6. If N/PN/NI to 2.3, or Y/PY/NI to 2.4 or 2.5: Was an appropriate analysis used to estimate the effect of adhering to the intervention?** |  | NA / Y / PY / PN / N / NI |
| **Risk-of-bias judgement** |  | Low / High / Some concerns |
| Optional: What is the predicted direction of bias due to deviations from intended interventions? | To us, we do not have to perform this part. Indeed, we stated at the beginning of the file that we focus on the effect of assignment to intervention | NA / Favours experimental / Favours comparator / Towards null /Away from null / Unpredictable |

Domain 3: Missing outcome data

| **Signalling questions** | **Comments** | **Response options** |
| --- | --- | --- |
| **3.1 Were data for this outcome available for all, or nearly all, participants randomized?** | The outcome was given in the findings section. | Y / PY / PN / N / NI |
| **3.2 If N/PN/NI to 3.1: Is there evidence that the result was not biased by missing outcome data?** | Not necessary | NA / Y / PY / PN / N |
| **3.3 If N/PN to 3.2: Could missingness in the outcome depend on its true value?** | Not necessary | NA / Y / PY / PN / N / NI |
| **3.4 If Y/PY/NI to 3.3: Is it likely that missingness in the outcome depended on its true value?** |  | NA / Y / PY / PN / N / NI |
| **Risk-of-bias judgement** | According to the algorithm, the result is ‘Low risk’ | Low / High / Some concerns |
| Optional: What is the predicted direction of bias due to missing outcome data? |  | NA / Favours experimental / Favours comparator / Towards null /Away from null / Unpredictable |

Domain 4: Risk of bias in measurement of the outcome

| **Signalling questions** | **Comments** | **Response options** |
| --- | --- | --- |
| **4.1 Was the method of measuring the outcome inappropriate?** | Physical examinations including assessment of performance status, (EORTC) European Organization for Reasearch and Treatment of Cancer, Quality of Life and laboratory evaluations, as well as ultrasonic studies, have been effectuated regularly, plus computed tomography (CT) and magnetic resonance imaging (MRI) when needed. Therapeutic benefit is assessed by time to progression and measuring quality of life. | Y / PY / PN / N / NI |
| **4.2 Could measurement or ascertainment of the outcome have differed between intervention groups?** | Evaluations were made with the same methods | Y / PY / PN / N / NI |
| **4.3 If N/PN/NI to 4.1 and 4.2: Were outcome assessors aware of the intervention received by study participants?** | No information has been given on this subject. | NA / Y / PY / PN / N / NI |
| **4.4 If Y/PY/NI to 4.3: Could assessment of the outcome have been influenced by knowledge of intervention received?** | No information has been given on this subject. | NA / Y / PY / PN / N / NI |
| **4.5 If Y/PY/NI to 4.4: Is it likely that assessment of the outcome was influenced by knowledge of intervention received?** |  | NA / Y / PY / PN / N / NI |
| **Risk-of-bias judgement** | According to the algorithm, the result is ‘High risk’ | Low / High / Some concerns |
| Optional: What is the predicted direction of bias in measurement of the outcome? | According to the algorithm the result is ‘High risk’. But in facts, we do not see a high risk here | NA / Favours experimental / Favours comparator / Towards null /Away from null / Unpredictable |

Domain 5: Risk of bias in selection of the reported result

| **Signalling questions** | **Comments** | **Response options** |
| --- | --- | --- |
| **5.1 Were the data that produced this result analysed in accordance with a pre-specified analysis plan that was finalized before unblinded outcome data were available for analysis?** | There is no data in the study on how the results were evaluated. The study only provided the following information: Interim data of the study document that in the MSC group no new metastases, neither hepatic nor other, have occurred, so far. On the contrary, several new metastases have developed in the control group. Conclusions: Orally administered MSC is a potent candidate to be regarded as a supportive therapy to surgery or plus chemotherapy for colorectal cancer patients.  Therefore, I answered no information to this group's questions. | Y / PY / PN / N / NI |
| **Is the numerical result being assessed likely to have been selected, on the basis of the results, from...** |  |  |
| **5.2. ... multiple eligible outcome measurements (e.g. scales, definitions, time points) within the outcome domain?** |  | Y / PY / PN / N / NI |
| **5.3 ... multiple eligible analyses of the data?** |  | Y / PY / PN / N / NI |
| **Risk-of-bias judgement** |  | Low / High / Some concerns |
| Optional: What is the predicted direction of bias due to selection of the reported result? |  | NA / Favours experimental / Favours comparator / Towards null /Away from null / Unpredictable |

Overall risk of bias

| **Risk-of-bias judgement** | **Overall risk of bias: High risk** | Low / High / Some concerns |
| --- | --- | --- |
| Optional: What is the overall predicted direction of bias for this outcome? |  | NA / Favours experimental / Favours comparator / Towards null /Away from null / Unpredictable |


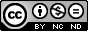


This work is licensed under a [Creative Commons Attribution-NonCommercial-NoDerivatives 4.0 International License](http://creativecommons.org/licenses/by-nc-nd/4.0/).

**NEWCASTLE - OTTAWA QUALITY ASSESSMENT SCALE**

**CASE CONTROL STUDIES**

Note: A study can be awarded a maximum of one star for each numbered item within the Selection and Exposure categories. A maximum of two stars can be given for Comparability.

**Selection**

1) Is the case definition adequate?

a) yes, with independent validation **🟑**

b) yes, eg record linkage or based on self reports

c) no description

2) Representativeness of the cases

a) consecutive or obviously representative series of cases **🟑**

b) potential for selection biases or not stated

3) Selection of Controls

a) community controls **🟑**

b) hospital controls

c) no description

4) Definition of Controls

a) no history of disease (endpoint) **🟑**

b) no description of source

**Comparability**

1) Comparability of cases and controls on the basis of the design or analysis

a) study controls for _______________ (Select the most important factor.) **🟑**

b) study controls for any additional factor **🟑** (This criteria could be modified to indicate specific control for a second important factor.)

**Exposure**

1) Ascertainment of exposure

a) secure record (eg surgical records) **🟑**

b) structured interview where blind to case/control status **🟑**

c) interview not blinded to case/control status

d) written self report or medical record only

e) no description

2) Same method of ascertainment for cases and controls

a) yes **🟑**

b) no

3) Non-Response rate

a) same rate for both groups **🟑**

b) non respondents described

c) rate different and no designation

**NEWCASTLE - OTTAWA QUALITY ASSESSMENT SCALE**

**COHORT STUDIES**

Note: A study can be awarded a maximum of one star for each numbered item within the Selection and Outcome categories. A maximum of two stars can be given for Comparability

**Selection**

1) Representativeness of the exposed cohort

a) truly representative of the average ______head and neck tumours (describe) in the community **🟑**

b) somewhat representative of the average ______________ in the community **🟑**

c) selected group of users eg nurses, volunteers

d) no description of the derivation of the cohort

2) Selection of the non exposed cohort

a) drawn from the same community as the exposed cohort **🟑**

b) drawn from a different source

c) no description of the derivation of the non exposed cohort

3) Ascertainment of exposure

a) secure record (eg surgical records) **🟑**

b) structured interview **🟑**

c) written self report

d) no description

4) Demonstration that outcome of interest was not present at start of study

a) yes **🟑**

b) no

**Comparability**

1) Comparability of cohorts on the basis of the design or analysis

a) study controls for _ no Avemar (fermented wheat germ) administration (select the most important factor) **🟑**

b) study controls for any additional factor **🟑** (This criteria could be modified to indicate specific control for a second important factor.)

**Outcome**

1) Assessment of outcome

a) independent blind assessment **🟑**

b) record linkage **🟑**

c) self report (sign &symptoms)

d) no description

2) Was follow-up long enough for outcomes to occur

a) yes (select an adequate follow up period for outcome of interest) **🟑**

b) no (there is no specific follow-up duration)

3) Adequacy of follow up of cohorts

a) complete follow up - all subjects accounted for **🟑**

b) subjects lost to follow up unlikely to introduce bias - small number lost - > __8,3__ % (select an adequate %) follow up, or description provided of those lost) **🟑** 5 patients could not survive. These patients were not included in the data analysis. 5/60

c) follow up rate < ____% (select an adequate %) and no description of those lost

d) no statement

**NEWCASTLE - OTTAWA QUALITY ASSESSMENT SCALE**

**(adapted for cross-sectional studies)**

**Selection:** (Maximum 3 stars)

1) Representativeness of the sample:

a) Truly representative of the average in the target population. **🟑** (all subjects or random sampling)

b) Somewhat representative of the average in the target population. **🟑** (non-random sampling)

c) Selected group of users.

d) No description of the sampling strategy.

2) Non-respondents:

a) Comparability between respondents and non-respondents characteristics is established, and the response rate is satisfactory. **🟑**

b) The response rate is unsatisfactory, or the comparability between respondents and non-respondents is unsatisfactory.

c) No description of the response rate or the characteristics of the responders and the non-responders.

3) Ascertainment of the exposure (risk factor):

a) Validated measurement tool. **🟑**

b) Non-validated measurement tool, but the tool is available or described.

c) No description of the measurement tool.

**Comparability:** (Maximum 2 stars)

1) The subjects in different outcome groups are comparable, based on the study design or analysis. Confounding factors are controlled.

a) The study controls for the most important factor (select one). **🟑**

b) The study control for any additional factor. **🟑**

**Outcome:** (Maximum 2 stars)

1) Assessment of the outcome:

a) Independent blind assessment. **🟑**

b) Record linkage. **🟑**

c) Self report.

d) No description.

2) Statistical test:

a) The statistical test used to analyze the data is clearly described and appropriate, and the

measurement of the association is presented, including confidence intervals and the probability level (p value). **🟑**

b) The statistical test is not appropriate, not described or incomplete.

This scale has been adapted from the Newcastle-Ottawa Quality Assessment Scale for cohort and case-control studies to perform a quality assessment of cross-sectional studies for the systematic review, “Exposure to second-hand smoke and the risk of tuberculosis in children and adults: systematic review and a meta-analysis of 18 observational studies”. This scale was a modified version of the NOS scale, as also used by several other studies that have felt the need to adapt the NOS scale so as to appropriately assess the quality of cross-sectional studies.

We did a comprehensive search on literature and found that a NOS score of 7 or more can be considered a “good” study (see McPheeters et al. 2012; see Appendix G page 103-104 in <http://www.ncbi.nlm.nih.gov/pubmedhealth/PMH0049229/>). So we used this criterion as a cut off for good quality study.

**NEWCASTLE - OTTAWA QUALITY ASSESSMENT SCALE**

**CASE CONTROL STUDIES**

Note: A study can be awarded a maximum of one star for each numbered item within the Selection and Exposure categories. A maximum of two stars can be given for Comparability.

**Selection**

1) Is the case definition adequate?

a) yes, with independent validation **🟑**

b) yes, eg record linkage or based on self reports

c) no description

2) Representativeness of the cases

a) consecutive or obviously representative series of cases **🟑**

b) potential for selection biases or not stated

3) Selection of Controls

a) community controls **🟑**

b) hospital controls

c) no description

4) Definition of Controls

a) no history of disease (endpoint) **🟑**

b) no description of source

**Comparability**

1) Comparability of cases and controls on the basis of the design or analysis

a) study controls for _______________ (Select the most important factor.) **🟑**

b) study controls for any additional factor **🟑** (This criteria could be modified to indicate specific control for a second important factor.)

**Exposure**

1) Ascertainment of exposure

a) secure record (eg surgical records) **🟑**

b) structured interview where blind to case/control status **🟑**

c) interview not blinded to case/control status

d) written self report or medical record only

e) no description

2) Same method of ascertainment for cases and controls

a) yes **🟑**

b) no

3) Non-Response rate

a) same rate for both groups **🟑**

b) non respondents described

c) rate different and no designation

**NEWCASTLE - OTTAWA QUALITY ASSESSMENT SCALE**

**COHORT STUDIES**

Note: A study can be awarded a maximum of one star for each numbered item within the Selection and Outcome categories. A maximum of two stars can be given for Comparability

**Selection**

1) Representativeness of the exposed cohort

a) truly representative of the average ______colorectal cancer patients (describe) in the community **🟑**

b) somewhat representative of the average ______________ in the community **🟑**

c) selected group of users eg nurses, volunteers

d) no description of the derivation of the cohort

2) Selection of the non exposed cohort

a) drawn from the same community as the exposed cohort **🟑**

b) drawn from a different source

c) no description of the derivation of the non exposed cohort

3) Ascertainment of exposure

a) secure record (eg surgical records) **🟑**

b) structured interview **🟑**

c) written self report

d) no description

4) Demonstration that outcome of interest was not present at start of study

a) yes **🟑**

b) no

**Comparability**

1) Comparability of cohorts on the basis of the design or analysis

a) study controls for _ no MSC (fermented wheat germ extract) administration(select the most important factor) **🟑**

b) study controls for any additional factor **🟑** (This criteria could be modified to indicate specific control for a second important factor.)

**Outcome**

1) Assessment of outcome

a) independent blind assessment **🟑**

b) record linkage **🟑**

c) self report

d) no description

2) Was follow-up long enough for outcomes to occur

a) yes (select an adequate follow up period for outcome of interest) **🟑**

b) no (there is no specific follow-up duration)

3) Adequacy of follow up of cohorts

a) complete follow up - all subjects accounted for **🟑**

b) subjects lost to follow up unlikely to introduce bias - small number lost - > __3,4__ % (select an adequate %) follow up, or description provided of those lost) **🟑** 6 patients of the control cohort started to take MSC on their own. These patients were not included in the data analysis. 6/176

c) follow up rate < ____% (select an adequate %) and no description of those lost

d) no statement

**NEWCASTLE - OTTAWA QUALITY ASSESSMENT SCALE**

**(adapted for cross-sectional studies)**

**Selection:** (Maximum 3 stars)

1) Representativeness of the sample:

a) Truly representative of the average in the target population. **🟑** (all subjects or random sampling)

b) Somewhat representative of the average in the target population. **🟑** (non-random sampling)

c) Selected group of users.

d) No description of the sampling strategy.

2) Non-respondents:

a) Comparability between respondents and non-respondents characteristics is established, and the response rate is satisfactory. **🟑**

b) The response rate is unsatisfactory, or the comparability between respondents and non-respondents is unsatisfactory.

c) No description of the response rate or the characteristics of the responders and the non-responders.

3) Ascertainment of the exposure (risk factor):

a) Validated measurement tool. **🟑**

b) Non-validated measurement tool, but the tool is available or described.

c) No description of the measurement tool.

**Comparability:** (Maximum 2 stars)

1) The subjects in different outcome groups are comparable, based on the study design or analysis. Confounding factors are controlled.

a) The study controls for the most important factor (select one). **🟑**

b) The study control for any additional factor. **🟑**

**Outcome:** (Maximum 2 stars)

1) Assessment of the outcome:

a) Independent blind assessment. **🟑**

b) Record linkage. **🟑**

c) Self report.

d) No description.

2) Statistical test:

a) The statistical test used to analyze the data is clearly described and appropriate, and the

measurement of the association is presented, including confidence intervals and the probability level (p value). **🟑**

b) The statistical test is not appropriate, not described or incomplete.

This scale has been adapted from the Newcastle-Ottawa Quality Assessment Scale for cohort and case-control studies to perform a quality assessment of cross-sectional studies for the systematic review, “Exposure to second-hand smoke and the risk of tuberculosis in children and adults: systematic review and a meta-analysis of 18 observational studies”. This scale was a modified version of the NOS scale, as also used by several other studies that have felt the need to adapt the NOS scale so as to appropriately assess the quality of cross-sectional studies.

We did a comprehensive search on literature and found that a NOS score of 7 or more can be considered a “good” study (see McPheeters et al. 2012; see Appendix G page 103-104 in <http://www.ncbi.nlm.nih.gov/pubmedhealth/PMH0049229/>). So we used this criterion as a cut off for good quality study.

**References**

Wells, G. A., Shea, B., O'Connell, D., Peterson, J., Welch, V., et al. The Newcastle-Ottawa Scale (NOS) for assessing the quality of nonrandomized studies in meta-analysis. 2011. <http://www.ohri.ca/programs/clinical_epidemiology/oxford.asp>

McPheeters, M.L., Kripalini, S., Peterson, N.B., Idowu, R.T. et al. (2012). Quality Improvement Interventions To Address Health Disparities. Evidence Report/ technology Assessment. Rockville (MD): Agency for Healthcare Research and Quality (US). <http://www.ncbi.nlm.nih.gov/pubmedhealth/PMH0049222/pdf/TOC.pdf>
